# Detection and assessment of electrocution in endangered raptors by infrared thermography

**DOI:** 10.1186/1746-6148-9-149

**Published:** 2013-07-23

**Authors:** Mar Melero, Fernando González, Olga Nicolás, Irene López, María de los Ángeles Jiménez, Susana Jato-Sánchez, José Manuel Sánchez-Vizcaíno

**Affiliations:** 1 VISAVET Centre and Animal Health Department, Veterinary School, Complutense University of Madrid, 28040 Madrid, Spain; 2 GREFA Native Fauna and Their Habitat Rehabilitation Group, 28220 Majadahonda, Spain; 3 Wildlife Rehabilitation Centre of Vallcalent, 25003 Lleida, Spain; 4 Medicine and Surgery Department (Anatomic Pathology), Veterinary School, Complutense University of Madrid, 28040 Madrid, Spain

**Keywords:** Thermography, Electrocution, Raptor, Bird of prey, Spanish imperial eagle, Lammergeier, Osprey

## Abstract

**Background:**

Most European birds of prey find themselves in a poor state of conservation, with electrocution as one of the most frequent causes of unnatural death. Since early detection of electrocution is difficult, treatment is usually implemented late, which reduces its effectiveness. By considering that electrocution reduces tissue temperature, it may be detectable by thermography, which would allow a more rapid identification. Three individuals from three endangered raptor species [Spanish imperial eagle (*Aquila adalberti*), Lammergeier (*Gypaetus barbatus*) and Osprey (*Pandion haliaetus*)] were studied thermographically from the time they were admitted to a rehabilitation centre to the time their clinical cases were resolved.

**Cases presentation:**

The three raptors presented lesions lacking thermal bilateral symmetry and were consistent with electrocution of feet, wings and eyes, visible by thermography before than clinically; lesions were well-defined and showed a lower temperature than the surrounding tissue. Some lesions evolved thermally and clinically until the appearance of normal tissue recovered, while others evolved and became necrotic. A histopathological analysis of a damaged finger amputated off a Lammergeier, and the necropsy and histopathology examination of an osprey, confirmed the electrocution diagnosis.

**Conclusions:**

These results suggest that thermography is effective and useful for the objective and early detection and monitoring of electrocuted birds, and that it may prove especially useful for examining live animals that require no amputation or cannot be subjected to invasive histopathology.

## Background

Birds of prey have been proposed as good sentinels of environmental changes as they are placed at the top of the food chain and are widespread worldwide [1]. However, 36 (64%) of the 56 species of raptors inhabiting Europe find themselves in an unfavourable state of conservation [2]. Most raptor deaths are caused by direct and indirect actions of humans [1]. One of the most common causes of unnatural death is electrocution as a result of collisions with power lines and the subsequent trauma when the animal falls to the ground [1,3,4]. While unnatural mortality can be compensated in a healthy population, it can seriously affect a small population [5]. For example, electrocution poses a significant survival problem for one of the most endangered raptors in the world, the Spanish imperial eagle [6].

The accuracy of electrocution mortality estimates is limited given the difficulty in determining cause of death [7]. Since determination of electrocution is usually based on anamnesis and clinical signs, i.e., electrical burns [1], detecting and evaluating an electrocuted bird can prove most difficult since anamnesis is often incomplete, and evidence for electrical trauma may not be immediately detectable [8]. Moreover, an early diagnosis of electrocution is essential for a good prognosis. For these reasons, complementary diagnostic tools, such as histopathology, are used when an amputation or necropsy is required [1,9]. Nevertheless, histopathology is invasive and may not be feasible or advisable for live animals, especially those that do not require amputation.

Thermography is a non-invasive technique used to assess tissue temperature that can be applied to electrocution detection and assessment. It can be used at a distance of more than 500 m from the animal [10], reducing its stress and ensuring the safety of the technician. Physiological and pathological thermographic patterns have not yet been established for most species. Nevertheless, reports of thermographic analyses have so far established that the thermal pattern and symmetry of the body are more important than the absolute temperature for diagnosing disease [11].

Tissue affected by electrocution shock shows reduced vascularisation, innervation, water content [12] and oxygen saturation [13]. These changes should result in a lower temperature in the affected area, thus raising the possibility that electrocution shock can be detected by thermography. Accordingly, this report applies thermography to detect and assess electrocution to three endangered raptors [14,15].

## Cases presentation

### Case 1: Spanish imperial eagle (*Aquila adalberti*)

On 11 September 2008, a juvenile female was admitted to the GREFA wildlife rehabilitation centre in Madrid (Spain). Clinical signs were 7% dehydration, depression, emaciation, arrhythmias, bradycardia and a proximal closed right radial fracture. Electrical lesions were not observed initially, but became visible 3 days later. Treatment included rehydration, antibiotics, a figure‒eight bandage on the right wing and physiotherapy on the right finger IV and the right wing. Finally, the animal was released on 16 March 2009.

**Figure 1:**
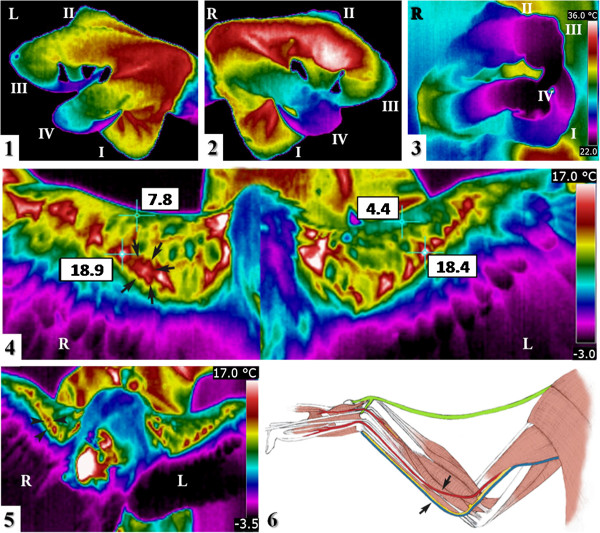
**Thermal analysis of the Spanish imperial eagle (*A. adalberti*). **Case 1. Thermography of feet; Lateral views of the **(1) **left foot and **(2) **the right foot upon admission, and **(3) **the right foot 35 days after admission. Roman numerals designate each finger (I-IV). A physiological thermal pattern is observed in the left foot and in right fingers I, II and III. The thermal range is the same for Figure 1(1-3). Thermography of wings; Ventral view of the wings taken **(4) **at the time the bandage was removed and **(5)** at the time of first flight test. A physiological thermal pattern is observed in the left wing. Initial designated right (R) and left (L) legs and wings. **(6) **An anatomic diagram of the ventral view of a right wing, drawn based on a figure in Orosz *et al*. [18]. The tendon of the tensor propatagialis is shown in green. The area of the proximal right radial fracture is enclosed by arrows **(4, 6)** and arrowheads **(5)**.

For thermographic evaluation (Cases 1–3), a ThermaCAM E45 infrared thermocamera with an FOV25 lens was used and images were analysed using the ThermaCAM QuickReport 1.2 SP2 software (FLIR, Burlington, ON, Canada). In this case, the thermal examination upon admission revealed an abnormally low temperature in right finger IV as compared to the other fingers, and a markedly higher temperature area in the right wing, which was coincident with the radius fracture (Table 1, Figure 1(1, 2)). During physiotherapy and rehabilitation, the thermal pattern of right finger IV and right wing became similar to that of the normal ones (Table 1, Figure 1(3)). Upon the first flight test, the eagle was unable to fly more than 5 seconds and deviated to the right and thermography of the right wing revealed an increase in the temperature of the muscles, especially in the elbow and carpus areas, probably due to the greater effort made by these less tone muscles in order to fly, as compared to the left wing, which presented the normal thermal pattern (Table 1, Figure 1(5)). For the remaining flight tests until the animal was released, the left and right wings showed the same thermal pattern.

**Table 1:** Thermal values at different anatomical areas

**Fingers**						
	**Healthy**		**Recovered**		**Required amputation**	
**Differences of temperature (°C)**	**Within each finger**	**Equivalent fingers of both feet**^**1**^	**Within each finger**	**Equivalent fingers of both feet**^**1**^	**Within each finger**	**Equivalent fingers of both feet**^**1**^
			**Right finger IV**			
**Spanish imperial eagle (Case 1)**	<5.0	0.2 - 1.5	Admission^2^ 6.6 Physio.^3^ 5.4	1.9 - 2.6		
			**Left finger IV**		**Left finger III**	
**Lammergeier (C 2)**	<3.6	<1.1	Admission^2^ 8.4 Physio.^3^ 3.7	6.1	10.2	8.4
**Legs**						
**Difference of temperature between legs (°C)**						
**Osprey (C 3)**	**Admission**			**Euthanasia**		
	4.1			13.6		
**Wings**						
			**Healthy (left wing)**		**Damaged (right wing)**	
**Temperature (°C)**			**Patagium area**	**Middle of the ulna area**	**Patagium area**	**Middle of the ulna area**
**Spanish imperial eagle (C 1)**	**Admission**		20.9	25.5	20.4	25.3
	**Removal of the bandage**		4.4	18.4	7.8	18.9
	**After the first flight test**		5.9	13.1	5.5	11.3
**Eyes**						
**Temperature (°C)**			**Left corneal temperature**	**Right corneal temperature**	**Intercorneal temperature difference**	
**Lammergeier (C 2)**	**17 April**		35.7	31.9	3.9^4^	
	**27 June**		34.2	33.5	0.7^4^	
**Six healthy lammergeiers**			29.8 - 32.3 (30.0, 29.8, 31.1, 30.6, 31.2, 32.3)	30.2 - 31.8 (30.2, 30.2, 31.5, 31.0, 31.0, 31.8)	0.2 - 0.5 (0.2, 0.4, 0.4, 0.4, 0.2, 0.5)	

^1^Differences of minimum temperature of equivalent fingers of both feet measured at the same phalange.

^2^Thermal measurement upon admission.

^3^Thermal values after physiotherapy treatment.

^4^ Difference above 0.6°C, defined by Morgan *et al*. [16] as the upper limit for 95% of the normal population. Analysed by a randomisation test [17], the differences between damaged and healthy eyes are statistically significant (p=0.036).

Cases 1–3. Thermographic measurements of healthy and damaged fingers, legs, wings and eyes of the three clinical cases and the eyes of six healthy Lammergeiers.

After removing the bandage, the thermography of the wings revealed a significantly higher temperature in the right patagium area (7.8°C) than in the left one (4.4°C) with a similar maximum temperature in the middle of both ulna areas (Table 1, Figure 1(4)). This asymmetric thermal pattern was consistent with the patagium retraction caused by periods of immobilisation. Increased vascularity and inflammation in the patagium area led to an increased temperature, which was detected by thermography. Early detection of this pathology is essential for a good prognosis; otherwise, the disease can lead to the development of fibrous tissue, calcifications and tie downs [19], making impossible the animal releasing into the wild, the primary goal of rehabilitation of wildlife.

### Case 2: Lammergeier (*Gypaetus barbatus*)

On 13 April 2012, an adult female was admitted to the fauna recuperation centre of Vallcalent, Lleida (Spain). The admission examination revealed proper body condition, good hydration and alert mental status. Nevertheless, the animal put little weight on the left limb and leaning instead on the dorsum of the foot. Treatment consisted of antibiotics, anti-inflammatory and physiotherapy on the left leg. The three most distal phalanges of left finger III were amputated 5 days after admission because their condition worsened despite treatment. The animal remained in the rehabilitation centre with a good prognosis, the amputated finger correctly healed and the left leg appeared strong and toned, and the animal was using it normally.

**Figure 2:**
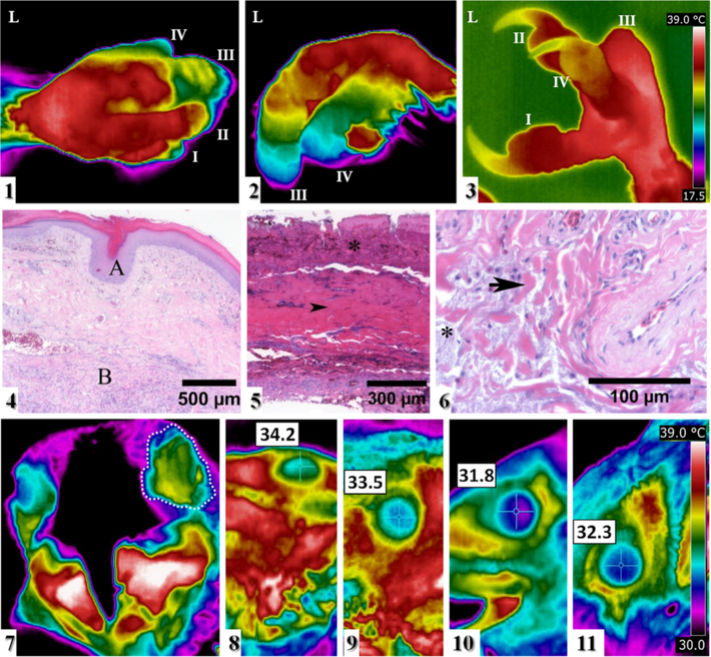
**Thermal analysis of the Lammergeier (*G***. ***barbatus*). **Case 2. **(1**–**3) **Thermal images of the left foot, taken **(1) **of the dorsal view and **(2) **of the lateral view on 17 April and **(3)** of the lateral view on 27 June. Initial designated left (L) leg and roman numerals designate each finger (I-IV). A physiological thermal pattern is observed in fingers I and II. **(4**–**6) **A histology section of left digit III stained with haematoxylin and eosin showing: **(4) **epidermis within normal limits (A) and deep dermis with broad linear segments of mixed inflammatory cells, oedema fluid and fragmented collagen fibres (B); **(5) **full thickness segmental coagulative necrosis with crushed keratinocyte nuclei (asterisk) and dermal collagenolysis (arrow head); **(6) **detail of mucinous material (asterisk) and smudged collagen fibres (arrow). Thermal images of eyes of two Lammergeiers. **(1**–**3) **Clinical case 2 before and during treatment: **(1) **Frontal view of the head taken on 17 April. The left eye is enclosed by a dotted line. A physiological thermal pattern is observed in the right eye. Lateral view of **(2) **the left eye and **(3) **the right eye, both taken on 27 June. **(4**,** 5) **Healthy Lammergeier, lateral view of **(4)** the left eye and **(5) **the right eye. The thermal range is the same for Figure 1**(1-3) **and for Figure 2**(7**-**11)**.

The first thermal examination showed an abnormal thermal pattern in left foot showing low temperatures and large temperature differences within finger III (10.2°C) and less marked in finger IV (8.4°C) compared with healthy ones (<3.6°C), an unusually high temperature and an irregular thermal pattern in the left eye and a significantly high difference in temperature between the centres of the two corneas (3.9°C) (Table 1, Figure 2(1, 2, 7). But after six weeks of treatment, the thermal pattern of the left finger IV and the left eye were normal and the temperature varied only 3.7°C in finger IV and the intercorneal temperature difference (0.7°C) was closer to that of eyes of healthy Lammergeiers (<0.6°C) (Table 1, Figure 2(3, 8-11)). Electrocution probably brought about the large intercorneal temperature difference and the loss of thermal structure in the left eye, and the deviations from bilateral symmetry decreased as the animal showed clinical improvement.

For the histology analysis (Cases 2, 3), tissue samples were fixed in formalin, lightly decalcified with nitrogen oxide when necessary, trimmed and paraffin-embedded. Tissues were then sectioned at a thickness of 4 μm and stained with hematoxylin-eosin (H-E) for diagnosis purposes. After the amputation, the histopathological examination of left finger III revealed broad segments of full thickness dermal coagulative necrosis (Figure 2(4-6)). Keratinocytes had bright eosinophilic homogeneous cytoplasm, fused cell borders and streaming basophilic nuclei. Broad linear multifocal segments of deep dermis, panniculus and tendon sheaths had brightly eosinophilic, glassy and fragmented collagen fibres, separated by clear space, mucinous material and scattered clusters of heterophils, lymphocytes, plasma cells and macrophages. Some arterioles had mural aggregates of similar inflammatory cells and glassy walls. These findings of severe subacute cutaneous avascular necrosis and heterophilic dermatitis were consistent with a third-degree burn. The segmental pattern of the full thickness coagulative necrosis and the lack of external lesions until sometime after the animal was admitted into rehabilitation were suggestive of an electrical rather than heat burn [20]. Electric current flows through areas of least resistance; hence, tissue is differentially affected [21,22].

Even before amputation, the thermography detected the abnormal thermal pattern and lack of thermal symmetry in left fingers III and IV, as well as the worse prognosis for finger III, thus supporting the idea that this non-invasive technique can gain useful clinical insights into affected animals.

### Case 3: Osprey (*Pandion haliaetus*)

On 16 March 2012, a juvenile male was admitted to GREFA. The clinical examination revealed an open, comminuted fracture of the left radius and ulna. Treatment included antibiotics, anti-inflammatories and propentofylline. On 24 March 2012, an intramedullary needle was surgically implanted into the left radius and ulna. After surgery, the animal’s condition continued to worsen; an oedema in the wing appeared and increased, and the animal could not stand up on its legs because of the neurovascular necrosis, which would have required amputation of the limb. Since amputation of the leg is incompatible with the hunting and feeding habits of this species, it was euthanised for humanitarian reasons on 28 March 2012 based on previous studies recommendations [23-25]. Then, necropsy was performed according to the protocol of Schmidt and Reavill [26,27].

**Figure 3:**
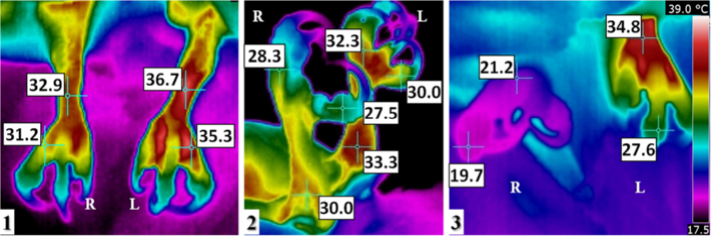
**Thermal analysis of the osprey (*P***. ***haliaetus*). **Case 3. Thermal images of legs. **(1) **Dorsal view and **(2)** right lateral view of the legs during the first examination. **(3) **Dorsal view on the day that euthanasia was practiced. Initial designated right (R) and left (L) legs. A physiological thermal pattern is observed in the left leg. The thermal range is the same for Figure 3**(1-3)**.

Thermal examination upon admission revealed an abnormally high difference between legs, which deteriorated significantly from admission (4.1°C) to the day that euthanasia was practiced (13.6°C) (Table 1, Figure 3) and a high temperature area in the left wing, from the elbow to the carpus, coincident with the fracture and surrounding tissue; and a low temperature in the region distally from the carpus. The comminute fracture and consequent inflammation increased the temperature between the elbow and the carpus, while the effect of electrocution lowered the temperature between the carpus and the distal part of the wing. During treatment the thermal pattern in this region become more marked and sharper boundaries with the surrounding tissue as a result of the animal’s worsened clinical status and of handling during surgery.

Necropsy revealed thinness, an oedema in the left wing, haematoma in ribs and keel, atrophied pectoral muscles, numerous clots distributed by all the vessels in lung and air sacs veins with yellowish deposits, thickened pericardium, myocardial greenish stain in the left ventricle, whitish injury in the apex, and liver yellowish and congestive encephalon. Cold oedemas mainly in the distal wing and coagulation in internal organs have been previously described as common necropsy findings in electrocuted birds [8]. The wing fracture and haematomas in pectoral muscles and keel were probably secondary to the fall after colliding with power lines.

The most remarkable histopathological findings were in the liver. The hepatic parenchyma had broad multifocal fragmented areas of coagulative necrosis surrounded by abundant immature granulation tissue and numerous foamy macrophages and giant multinucleated cells. The viable hepatic parenchyma adjacent to the granulation tissue was disorganized and contained numerous packed small groups of oval cells arranged in tubules (regeneration) and separated by moderate amounts of mature fibrous tissue, haemosiderin and bile-laden macrophages, heterophils and lymphocytes. The hepatic coagulative necrosis was suggestive of blunt trauma, often associated with electrocution [28-30]. Tissue is damaged due to the direct effects of the electrical current as it dissipates through organs [29,30]. Moreover, as this injured animal was located close to power lines, electrocution was highly suggestive.

The anamnesis, the veterinary examinations, and the histopathological analyses and necropsies, when performed, confirmed electrocution of these three animals. Thermographically, temperature reduction caused by electrocution was detected since the animals’ admission, and prior to macroscopic lesions appearing. Additionally, the comparison made of the thermographs before and after physiotherapy revealed that thermography may help predict tissue damage prognosis and guide rehabilitation efforts.

Thermographically, injured tissues were cooler than the corresponding healthy ones and the thermal pattern was altered, which showed that thermal symmetry and thermal distribution are more important parameters than absolute temperature. Mabuchi *et al*. [11] drew the same conclusion when assessing the application of thermography for clinical diagnoses in humans. However, as reference absolute values are currently lacking, each clinical case should be studied carefully by performing thermography before and during rehabilitation in order to follow the thermal evolution. Although thermography is the most rapid and non-invasive tool for electrocution diagnosis, histopathology should be used to confirm the suspicious cases.

## Conclusions

Thermography is a rapid, stress-free and objective tool that allows: the early detection of asymmetry temperature distribution and lesions consistent with electrocution without necropsy or amputation; the determination of areas damaged by the entry and exit of an electric current; the assessment of the animal’s clinical status; the diagnosis of pathologies associated with electrocution, such as fractures and patagium retraction; and the evaluation of how lesions evolve with different treatments; all of which can contribute to more effective treatment.

## Competing interests

The authors declare that they have no competing interests.

## Authors’ contributions

MM contributed by taking the thermograms and images analysis and by writing the manuscript. FG, ON, IL, MAJ and SJS helped draft the manuscript. FG, ON and IL contributed with the clinical diagnosis, treatments and assessments. MAJ contributed by performing the histopathology analysis. SJS contributed with the physiotherapy diagnosis and treatment at GREFA. JMSV contributed to coordinating and reviewing the whole process. All the authors have read and approved the final manuscript.
